# Residents’ participation in energy performance certification for collective action on climate change: the case of EnerGuide

**DOI:** 10.3389/fpsyg.2023.1196208

**Published:** 2023-07-11

**Authors:** Xinyu Chen, Zhonghua Gou, Huihua Zhang

**Affiliations:** ^1^School of Urban Design, Wuhan University, Wuhan, China; ^2^School of Entrepreneurship, Jingdezhen Ceramic University, Jingdezhen, Jiangxi, China

**Keywords:** climate change collective action, residential energy efficiency, energy performance certification, climate policy, energy policy

## Abstract

**Introduction:**

Although governments have launched energy performance certification (EPC) programs to increase residential energy efficiency, residents’ perception and acceptance of these programs have been little studied. This study contextualizes residents’ intentions to participate in EPC programs in terms of their attributions to and attitudes toward climate change to find mediating factors and effects to help trigger collective action to reduce residential sector energy demand.

**Methods:**

This study employed a partial least squares structural equation modeling approach to empirically analyze a survey conducted on 400 residents of Edmonton, Canada, who participated in the Canadian energy efficiency rating and labeling program, EnerGuide.

**Results and Discussion:**

Using EnerGuide, a Canadian energy efficiency rating and labeling program, as an example, this study establishes a framework to explain that local residents’ internal and external attributions to climate change elicit positive attitudes (need to take action), increasing their recognition of energy efficiency program benefits, which further promotes their EnerGuide program acceptance and participation intentions. This study also reveals the mediating effects between variables. Residents’ attitudes toward climate change mediate the relationship between internal/external attributions and EnerGuide program acceptance, and they indirectly impact residents’ program acceptance and participation intentions, with this effect moderated by energy efficiency program benefits. Residents’ program acceptance also mediates the relationship between climate change attitudes/energy efficiency program benefits and the intention outcome.

**Implication:**

The study provides an example of the use of climate change discourse to motivate residents’ energy efficiency program participation.

## 1. Introduction

Despite overwhelming scientific evidence and a comprehensive set of policy instruments, concrete actions on climate change remain vague, and cooperation across the various sectors is lacking. Admittedly, climate change has become a collective action problem, and research is needed to understand the public’s perceptions, attitudes, benefits, acceptance, and intentions to act on climate-related issues and solutions (Coglianese, 2022). This study takes the residential sector, an important global energy end-user, as an entry point to solve the collective action dilemma in response to climate change. Despite improvements in building design and construction techniques and the accelerating deployment of renewable technologies, the total energy use demand in residential buildings has continued to grow (International Energy Agency, 2019, 2021). Additionally, many households are facing a growing cost of living crisis as global energy (such as oil and natural gas) prices continue to rise sharply. Improving energy efficiency in the residential sector has become a focus of governments worldwide seeking to ease the pressure from both the climate and energy crises (International Energy Agency, 2017, 2022). Research on residents’ perceptions and participation in energy efficiency programs as collective action on climate change holds great significance for governments to formulate two-pronged policies to tackle the crises facing society today.

As one of the most effective measures, building energy performance certification (EPC) aims to provide policymakers and relevant stakeholders with “how-to” guidance on the essential elements of implementing a building EPC program (International Energy Agency, 2010). Building EPC is a key policy instrument that can not only help governments reduce building energy consumption and alleviate the energy crisis but also raise residents’ awareness of energy consumption in their daily lives and reduce household energy expenditures. To date, building EPC programs have been launched in various regions around the world. They are a rating scheme that summarizes the energy efficiency of buildings, and they play an important role in retrofitting existing buildings into near-zero energy buildings to meet the decarbonization agendas of governments, as they provide transparent information on energy performance. For example, ENERGY STAR is a government program jointly launched by the U.S. Department of Energy (DOE) and the U.S. Environmental Protection Agency (EPA) to improve energy efficiency by providing information on residential energy consumption. Specifically, ENERGY STAR-certified homes are at least 10% more energy efficient than homes built to code (Energy star, 2021). In the European region, EPCs were first introduced in the Energy Performance of Buildings Directive (EPBD) in 2002 (European Union, 2002), and the EPBD was revised in 2010 with a series of new requirements to improve quality, availability and public acceptance (European Union, 2010). An EPC usually includes a label that provides residents with the building’s energy performance rating, general information about the building (age, location, etc.), and expert advice on how to improve the building’s energy efficiency (Zuhaib et al., 2022).

**Figure 1:**
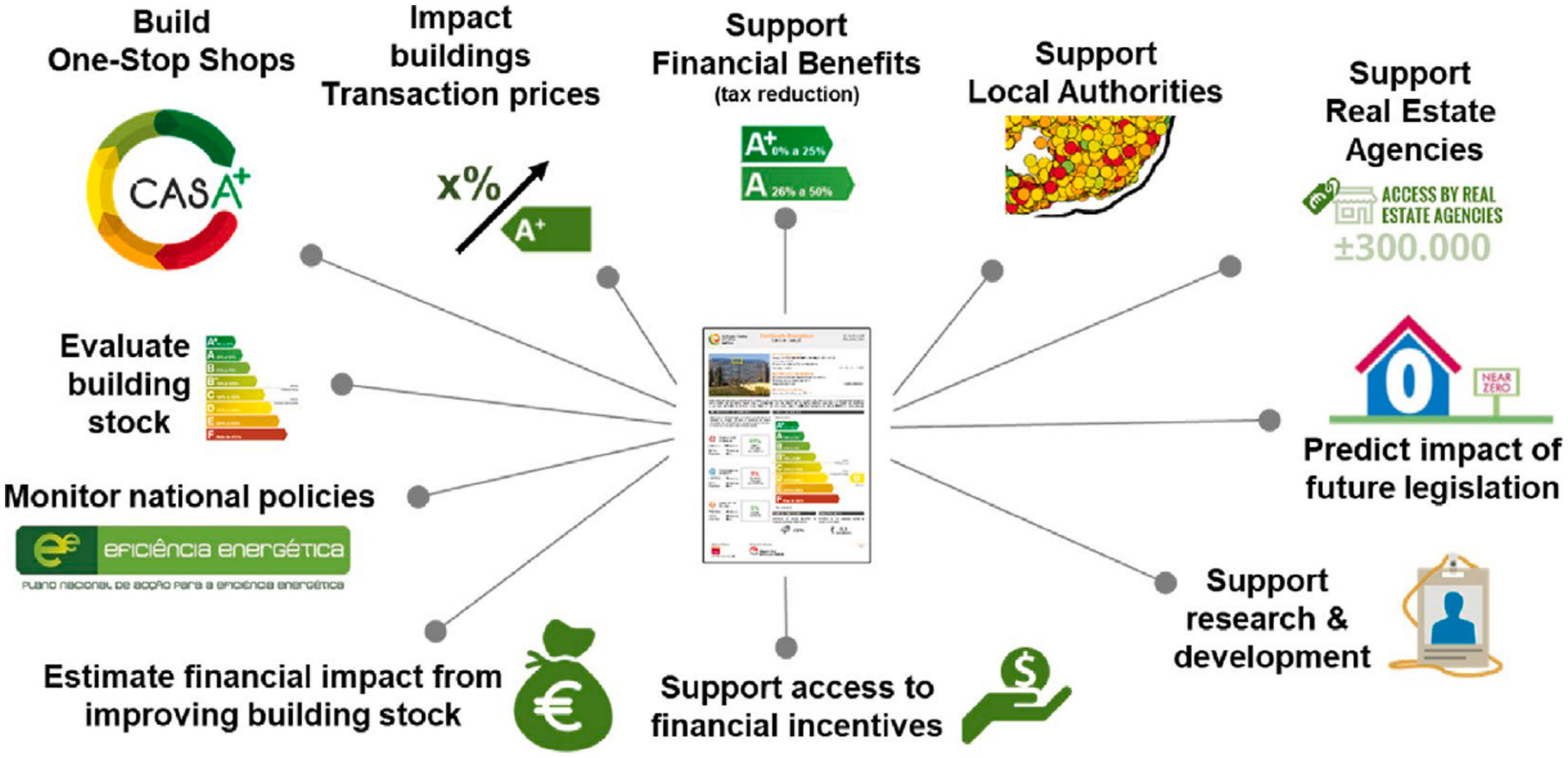
Potential functions and roles of EPCs (Volt et al., 2020).

Arguably, EPC programs not only can be powerful instruments for conserving energy and reducing greenhouse gas (GHG) emissions in the residential sector but also give policymakers access to better building stock data and enable them to effectively monitor the impact of policies in the process of implementation. Figure 1 shows some of the functions enabled by EPCs. EPCs have been used in decision support planning to encourage homeowners to renovate buildings (Chegut et al., 2014), they and have also been used as a major tool to promote the decarbonization of the building stock (Li et al., 2019). For example, the UK, France and the Netherlands use EPCs to set mandatory minimum energy performance standards for existing buildings (Volt et al., 2020). However, in other countries, such as Germany, EPC programs have not been widely welcomed (Amecke, 2011). The wider use of EPCs and their information includes support for local governments, real estate agencies, and academic research as well as the development of urban energy policy (Pasichnyi et al., 2019). In recent years, existing research has mainly focused on revealing the usefulness and reliability of EPCs. Although several studies have highlighted the limited impact of EPCs on homeowners’ energy retrofitting practices and purchasing decisions (Amecke, 2012; Christensen et al., 2014), many researchers and industry experts have argued that EPCs can help overcome the challenges associated with housing decarbonization, deep retrofitting, the development of recommendations, future energy savings and overall sustainability (von Platten et al., 2019; Wilhelmsson, 2019; Khazal and Sønstebø, 2020; Anđelković et al., 2021). Admittedly, there are challenges to achieving mass acceptance of EPCs on a global scale. Some important reasons are that the information provided to motivate homeowners to participate in EPCs and to retrofit their buildings is insufficient, and in some countries, there is limited implementation to provide a reliable source of information for home energy planning, which is required by EPCs (Christensen et al., 2014; Mangold et al., 2015).

Despite the promising role of EPCs in the decarbonization of the building sector, low public participation handicaps the large-scale implementation and execution of EPCs (Schuitema et al., 2020). The way out of this dilemma requires further understanding the public’s perceptions, attitudes, benefits, acceptance, and intentions to act. This study contextualizes residents’ benefits, acceptance and participation intentions in terms of the discourse of climate change, with the aim of understanding whether climate change attributions and attitudes influence residents’ recognition of the benefits of energy efficiency programs and acceptance of EPC programs and further motivate or discourage their participation. Collective action on climate change requires a clear pathway out of the dilemma to make substantial progress in carbon-intensive sectors such as the residential energy sector. Using EnerGuide, a Canadian energy efficiency rating and labeling program, as an example, this research can provide such a clear pathway for combining climate policies and energy policies in the residential sector as a two-pronged approach to tackling the crises facing society today.

## 2. Research framework

A growing body of research on pro-environmental intentions and behaviors makes extensive use of the theory of planned behavior (TPB) (Ajzen, 1991) and value-belief-norm (VBN) theory (Stern, 2000). The main strengths of these two theories lie in investigating and understanding the motivational foundations behind individuals’ engagement or nonengagement in environmentally significant actions. The TPB can help us understand how people change their behavioral patterns. The theory states that attitudes, subjective norms, and perceived behavioral control together shape individuals’ behavioral intentions and behaviors. Furthermore, VBN theory, which is used to study environmentally important behaviors, postulates that values influence pro-environmental behaviors through pro-environmental beliefs and personal norms. Although these two theories have been widely used in the study of pro-environmental behavior and have been examined and supported by a large number of empirical studies (Chen, 2016; Li et al., 2017; Chen and Gou, 2022), they have some shortcomings. These two models have been used to test people’s general views on climate or ecological crises, but information related to collective action on climate change explored in this study, such as individuals’ cognition of climate change issues, perception of the benefits of energy efficiency programs, and extent of acceptance of EPC programs, is lacking. These additional variables lay the foundation for residents’ intentions to participate in the EnerGuide program. Therefore, this study establishes a framework that contextualizes the acceptance of and participation in EPC programs in terms of climate change discourse to meet the objective of this research. The key variables and their measurements are explained as follows.

### 2.1. Attributions of climate change

Attribution theory (AT) concerns a cognitive process by which people analyze their own or others’ behaviors and infer the reasons for these behaviors (Heider, 1958; Ryan and Connell, 1989). In recent years, a large number of studies have shown that public awareness of the attribution of climate change problems is increasing (Ogunbode et al., 2019, 2020). At the same time, people with a high cognitive need for information on climate issues will seek and obtain such information, thereby forming stronger and more stable attitudes (Barbaro et al., 2015). Understanding individuals’ cognition of attribution promotes citizens’ attitudes and behavioral intentions toward climate action, and it has a positive impact on climate solutions, such as improving residential energy efficiency (Guo et al., 2021; Lin et al., 2021).

An attribution may be caused by factors beyond the individual’s control (external), or an attribution to the individual’s own behavior (internal) may be made (Garg et al., 2021). Internal attribution is the act of attributing responsibility to certain types of factors or criteria that can be controlled by an individual. The public generally believes that the main cause of climate change is human actions rather than nature. Therefore, human attitudes toward climate change are closely related to individuals’ attribution of climate change (Mo Jang, 2013; Wong-Parodi and Rubin, 2022). At the same time, the behavioral intention to take climate action is also influenced by external factors beyond individuals’ attribution (Mainieri et al., 1997; Azucena et al., 2013; Blok et al., 2015). External attribution is individuals’ belief that the good and bad factors in their lives are controlled by forces and circumstances that are external to themselves. This is also called external locus of control. Studies have shown that external environmental locus of control is a significant positive predictor of perceived pro-environmental attitudes and has a partial mediating effect (Giefer et al., 2019; Sharma et al., 2022). At the same time, external attribution has a positive impact on pro-environmental acceptance (Lois et al., 2016).

### 2.2. Attitudes toward climate change

Attitude is a psychological structure that refers to an individual’s positive or negative evaluation of a particular object, such as a person, an idea, an emotion, or an event (Eagly and Chaiken, 1993). Attitudes are a core component of the theory of reasoned action (TRA) and the TPB, and they shape an individual’s behavioral intentions (Chen et al., 2017; Duarte et al., 2017). Prete et al. (2017) argued that attitudes are the main determinant driving households’ intentions to implement energy efficiency measures. After an extensive literature review, we found that environmental attitudes are directly related to behavioral intentions and contribute to the development of pro-environmental behaviors (Iozzi, 1989; Mamun et al., 2018; Ateş, 2020; Masukujjaman et al., 2021). In addition, attitudes have significant positive effects on the benefits and acceptance of pro-environmental behaviors (Tarigan and Bayer, 2012; Chang, 2014). For example, Craig (2018) found that attitudes can influence residents’ likelihood of supporting policies for energy efficiency subsidies provided by utility organizations.

### 2.3. Recognition of benefits

In addition to the general perception of climate change, the benefits of environmentally friendly or energy efficiency programs have a positive role in motivating people’s acceptance and participation, which has been confirmed in research on nuclear power (Visschers et al., 2011), home energy efficiency improvements (Cole et al., 2018), and waste sorting practices (Cudjoe et al., 2020). Individuals thoroughly evaluate the perceived utility of their behavior prior to performing a specific behavior (Zeithaml, 1988). Researchers and theorists believe that behavior is driven by an individual’s cognitions of acceptability, motivation, and attitudes toward the behavior, especially in positive contexts (Yazdanpanah et al., 2014; Adu-Gyamf et al., 2022). The benefits of environmentally friendly or energy efficiency programs such as EPCs refer to the perception of the positive consequences of participation in these programs and are favorable assumptions or beliefs about the outcomes of behavioral participation (Cudjoe et al., 2020).

**Figure 2:**
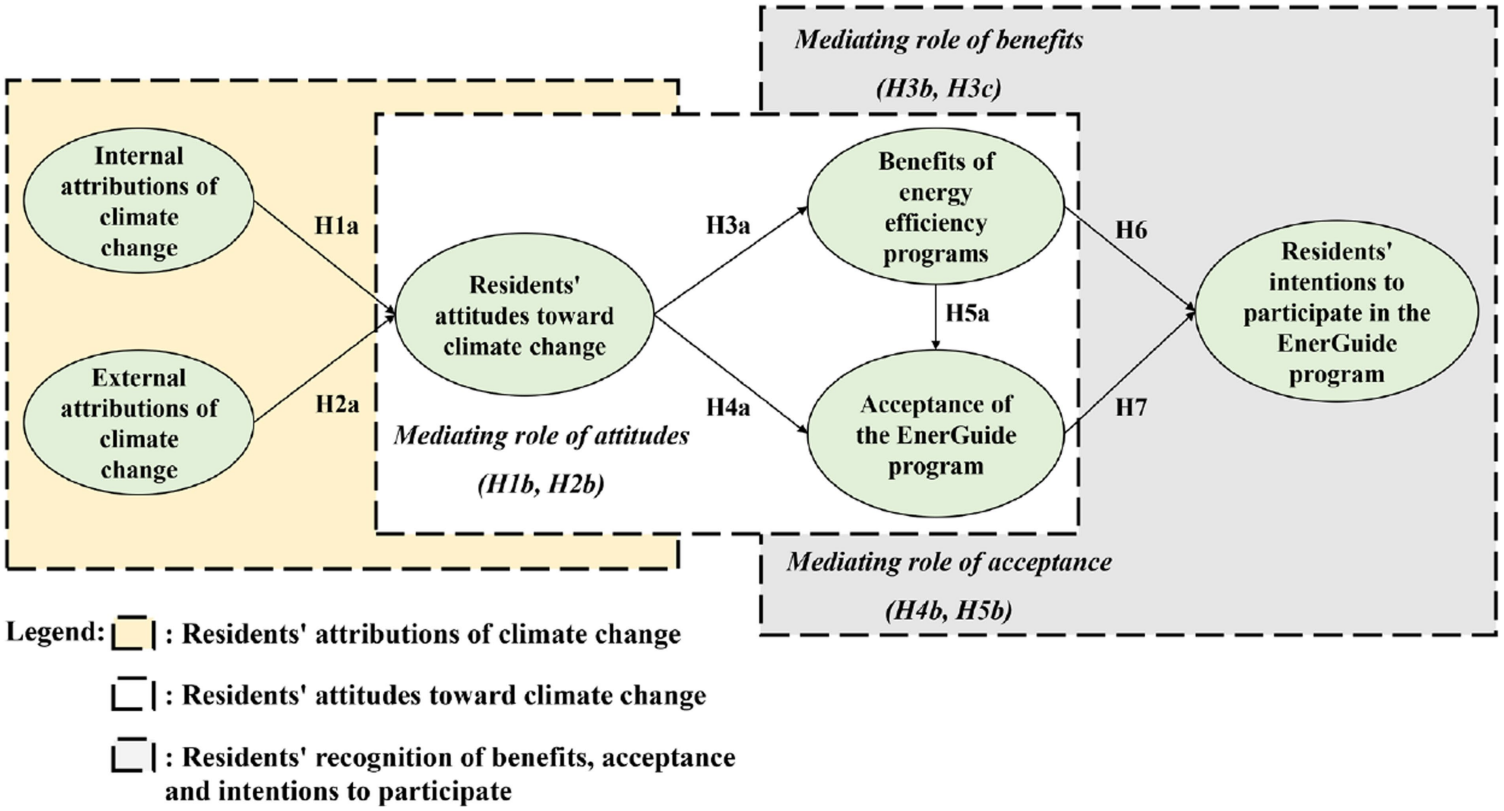
Conceptual framework of this study.

Based on the literature review above, this study infers that internal and external attributions of climate change, residents’ attitudes toward climate change, and recognition of the benefits of energy efficiency have a positive impact on improving residents’ acceptance of the EnerGuide program and their intentions to participate in this program. The conceptual framework to be tested is shown in Figure 2, and the specific hypotheses are presented in Table 1.

**Table 1:** Hypotheses to be tested.

Items	Hypotheses
H1a	Internal attributions of climate change are positively correlated with attitudes toward climate change.
H1b	Attitudes toward climate change have a mediating effect on the relationship between internal attributions of climate change and acceptance of the EnerGuide program.
H2a	External attributions of climate change are positively correlated with attitudes toward climate change.
H2b	Attitudes toward climate change have a mediating effect on the relationship between external attributions of climate change and acceptance of the EnerGuide program.
H3a	Attitudes toward climate change are positively correlated with the benefits of energy efficiency programs.
H3b	The benefits of energy efficiency programs have a mediating effect on the relationship between attitudes toward climate change and acceptance of the EnerGuide program.
H3c	The benefits of energy efficiency programs have a mediating effect on the relationship between attitudes toward climate change and intentions to participate in the EnerGuide program.
H4a	Attitudes toward climate change are positively correlated with acceptance of the EnerGuide program.
H4b	Acceptance of the EnerGuide program has a mediating effect on the relationship between attitudes toward climate change and intentions to participate in the EnerGuide program.
H5a	The benefits of energy efficiency programs are positively correlated with acceptance of the EnerGuide program.
H5b	Acceptance of the EnerGuide program has a mediating effect on the relationship between the benefits of energy efficiency programs and intentions to participate in the EnerGuide program.
H6	The benefits of energy efficiency programs are positively correlated with intentions to participate in the EnerGuide program.
H7	Acceptance of the EnerGuide program is positively correlated with intentions to participate in the EnerGuide program.

## 3. Methodology

### 3.1. EnerGuide program

The EnerGuide program is the main energy rating and labeling system used in Canada to certify the energy efficiency of major consumer products (houses, light vehicles, and certain energy-using products) (Government of Canada, 2020). EnerGuide ratings allow residents to easily compare the energy efficiency of major home appliances sold in Canada. In addition, home energy assessments are an important part of the EnerGuide program, which includes home assessments, labels indicating home energy performance and expert advice on how to improve energy efficiency.

Additionally, cities offer a $400 discount on fees to help residents pay for assessments, which typically cost between $400 and $800. Since 2016, the EnerGuide rating system has changed. The new EnerGuide tab shows the gigajoules (GJ, the annual unit of energy measurement) that a house uses each year. The closer the annual GJ measurement is to zero, the more efficient the house is. Compared with other EPC programs such as ENERGY STAR in the U.S. and the EPBD in Europe, the EnerGuide is less investigated. This study on EnerGuide not only enriches the literature on residential energy efficiency but also provides a good example for developing countries such as China and India to learn from.

### 3.2. Data collection

The data used in this study were obtained from the Edmonton government’s open data website (City of Edmonton, 2018). Edmonton is the capital city of Alberta, Canada. As of 2021, the City of Edmonton had a metropolitan population of 1,418,118, making it Canada’s fifth-largest city (Statistics Canada, 2022) and sixth-largest census metropolitan area (CMA) (Statistics Canada, 2017). From June 18 to June 24, 2018, the City of Edmonton conducted a city-wide survey on climate change and energy perceptions among residents aged 18 or older. A total of 1,000 residents participated in the online questionnaire.

The City of Edmonton -–2018 Climate Change and Energy Perceptions survey covers a series of questions (the complete questionnaire and data are shown in Supplementary Appendix A). Based on the conceptual framework proposed in this study (Figure 2), we selected 20 measurement items from the original questionnaire that are relevant for testing the specific meanings of each social psychological indicator in the framework (see Supplementary Appendix B: Table B.1) to adapt to the context of the current research. In addition, to ensure the accuracy of this study, the responses of 600 individuals were removed from the dataset because they had marked “Not Sure” or responded incompletely on all items of one or more factors. Finally, a total of 400 responses were included for empirical analysis. In the climate change discourse framework, the key attribution items include individuals’ internal and external attributions of climate change. The attitude items mainly involve individuals’ attitudes toward climate change. The benefit items include individuals’ recognition of the benefits of energy efficiency programs. The acceptance items mainly involve individuals’ acceptance of the EnerGuide program, and the intention items cover individuals’ intentions to participate in the EnerGuide program. The 20 items were scored using a 5-point Likert scale. In addition, to ensure the consistency of the answer options for all items, measures of internal attributions, external attributions, residents’ attitudes toward climate change, the benefits of energy efficiency programs, acceptance of the EnerGuide program and residents’ intentions to participate in the EnerGuide program were preprocessed for this study before testing the research hypotheses, with answers to all items ranging from 1 (“strongly disagree”) to 5 (“strongly agree”). We also performed reliability analyses for each construct. The Cronbach’s alpha (CA) values for all constructs were found to be greater than the suggested value of 0.70, indicating good internal consistency (Hair, 2004; Hinton, 2004).

### 3.3. Descriptive statistics of the respondents

The basic information of the respondents is generally consistent with the local census profile (Statistics Canada, 2022). As shown in Supplementary Appendix C: Table C.1, 209 males (52.25%) and 191 females (47.75%) responded to the survey. In terms of age, 18.50% of the respondents were aged 25–34, 18.75% were aged 35–44, 17.50% were aged 45–54, 23.75% were aged 55–64, and 19.00% were aged 65 and above. Regarding annual household income before tax, more than 50% of the respondents had an annual household income before tax of more than $80,001. In terms of the highest degree of education, nearly 50% of the respondents had at least an undergraduate degree. With regard to the housing type, 72.50% of the respondents had their own completely independent houses.

### 3.4. Data analysis techniques

To evaluate the proposed research hypotheses and research model, this paper uses SmartPLS 3.2.0 software to perform partial least squares structural equation modeling (PLS-SEM). PLS-SEM is a component-based estimation method (Wold, 1982; Lohmöller, 1989) that is mainly suitable for interpreting complex relationships (Liang et al., 2007), dealing with formative structures (Chin et al., 2003) and analyzing nonnormally distributed data (Hair et al., 2017). The PLS-SEM structural equation model consists of two parts: the measurement model and the structural model (Anderson and Gerbing, 1988). The measurement model represents the relationship between the observed data and the latent variables (internal attributions, external attributions, residents’ attitudes toward climate change, the benefits of energy efficiency programs, acceptance of the EnerGuide program, and residents’ intentions to participate in the EnerGuide program). The structural model represents the relationships between latent variables.

**Figure 3:**
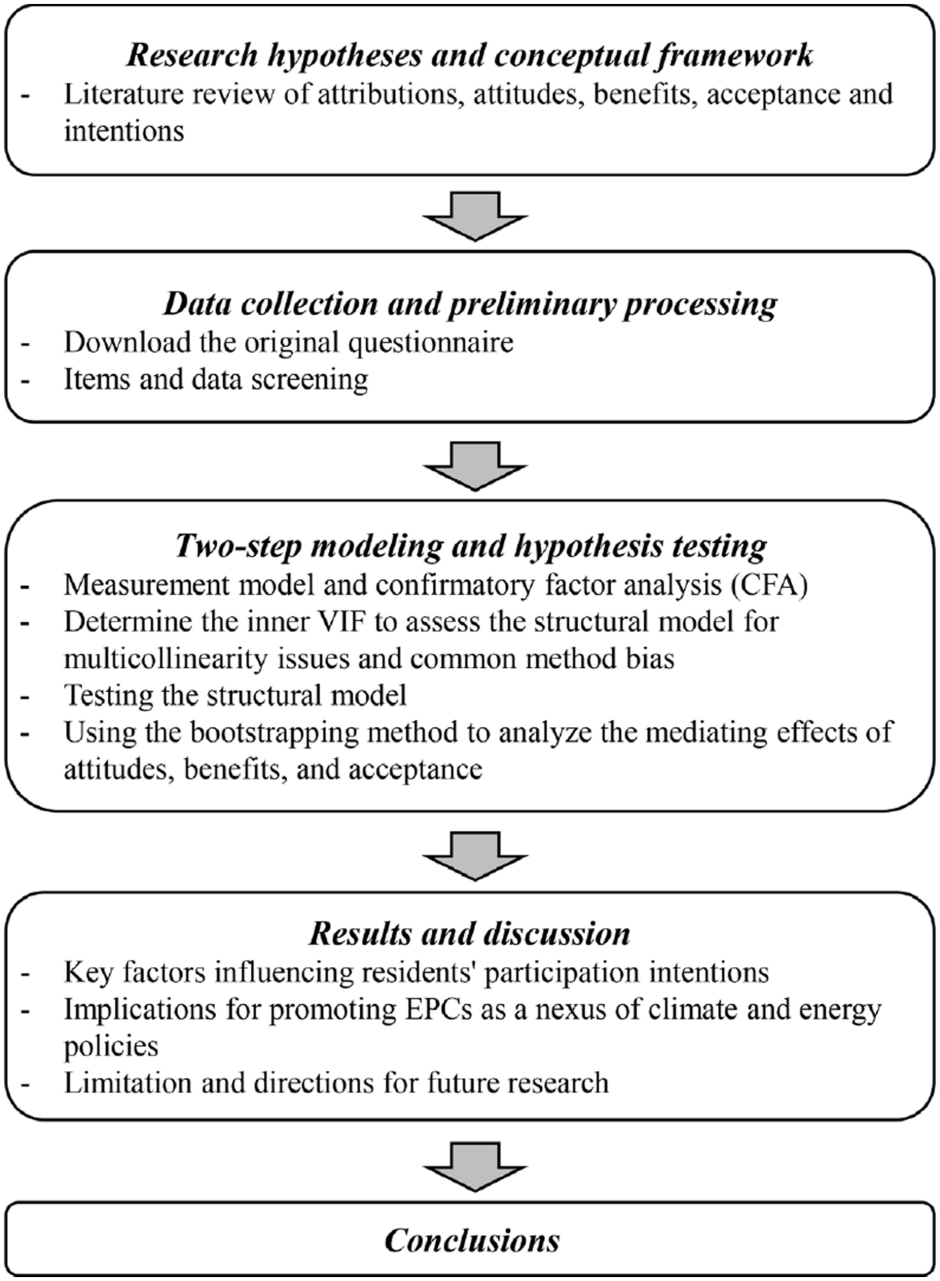
Flowchart of the data analysis.

Figure 3 shows the overall analysis steps in this paper. First, confirmatory factor analysis (CFA) was used to evaluate the reliability and validity of the measurement model. In addition, the Fornell–Larcker criterion (Fornell and Larcker, 1981) was used to test the discriminant validity of all constructs. Second, before testing the structural model, we assessed whether the structural model has multicollinearity problems and common method bias by determining the inner variance inflation factor (VIF). Finally, the structural model was evaluated to validate the hypotheses of this study. Again, the mediating effects of residents’ attitudes toward climate change, residents’ recognition of the benefits of energy efficiency programs, and residents’ acceptance of the EnerGuide program were tested (using the bootstrapping method).

## 4. Results

### 4.1. Evaluation of the measurement model

CFA was used to assess the reliability and validity of the measurement model. As shown in Table 2, the outer loading for all items met the benchmark of 0.70 (Hair et al., 1998). To evaluate the reliability of the constructs, this study used CA values and composite reliability (CR) values to evaluate the internal consistency reliability of all constructs (Fornell and Larcker, 1981). Table 2 shows that the CA values (0.735–0.944) and CR values (0.851–0.960) of all constructs of the measurement model were higher than the suggested value of 0.70. Since constructs with high internal consistency typically have highly correlated metrics, this suggests that the reliability of all constructs was supported (Hair et al., 1998). In addition, the loading values and average variance extracted (AVE) values (0.658–0.857) of all constructs met the threshold values recommended by Hair et al. (2017). Therefore, all constructs showed sufficient evidence of convergent validity.

**Table 2:** Measurement items.

Constructs	Items	Loadings	CA	CR	AVE
Internal attributions of climate change	IACH1	0.869	0.828	0.897	0.744
	IACH2	0.905			
	IACH3	0.811			
External attributions of climate change	EACH1	0.914	0.910	0.943	0.847
	EACH2	0.926			
	EACH3	0.921			
Attitudes toward climate change	ATCH1	0.922	0.944	0.960	0.857
	ATCH2	0.930			
	ATCH3	0.919			
	ATCH4	0.932			
Benefits of energy efficiency programs	BEEP1	0.910	0.910	0.943	0.847
	BEEP2	0.922			
	BEEP3	0.929			
Acceptance of the EnerGuide program	AEGP1	0.911	0.925	0.947	0.817
	AEGP2	0.898			
	AEGP3	0.897			
	AEGP4	0.908			
Intentions to participate in the EnerGuide program	IPEGP1	0.850	0.735	0.851	0.658
	IPEGP2	0.862			
	IPEGP3	0.713			

This study also assessed the discriminant validity of all constructs using the Fornell–Larcker criterion (Fornell and Larcker, 1981). The Fornell–Larcker criterion requires that the square root of the AVE value for each construct be higher than the link between all constructs. Therefore, as shown in Table 3, the discriminant validity of all constructs of the measurement model is satisfactory.

**Table 3:** Discriminant validity.

Constructs	IACH	EACH	ATCH	BEEP	AEGP	IPEGP
Internal attributions of climate change	**0.862**					
External attributions of climate change	0.782	**0.920**				
Attitudes toward climate change	0.846	0.745	**0.926**			
Benefits of energy efficiency programs	0.728	0.721	0.744	**0.920**		
Acceptance of the EnerGuide program	0.612	0.637	0.631	0.660	**0.904**	
Intentions to participate in the EnerGuide program	0.538	0.518	0.593	0.560	0.693	**0.811**

The square root of the AVE value is shown in bold.

### 4.2. Evaluation of the structural model (hypothesis testing results)

Before testing the structural model, to examine common method bias in the empirical results, we adopted the studies of Podsakoff et al. (2003) and Kock (2015). First, the City of Edmonton – 2018 Climate Change and Energy Perceptions Survey included background information, introductory information, and a detailed description of the questions to minimize uncertainties in the questionnaire. In addition, the responses of all respondents were anonymous, and the survey indicated that there were no right or wrong answers (MacKenzie and Podsakoff, 2012). Second, we checked whether the structural model had collinearity problems, i.e., whether the inner VIF values were less than the recommended threshold of 3.3 (Petter et al., 2007). The results of this study showed that the inner VIF values of all independent variables were less than the benchmark value of 3.3. Therefore, not only did the structural model of this study show no signs of multicollinearity, but common method bias was also not a serious problem.

This study used SmartPLS 3.2.0 to evaluate the hypothetical model. The analysis results showed that the adjusted *R*^2^ of residents’ attitudes toward climate change, residents’ recognition of the benefits of energy efficiency programs, residents’ acceptance of the EnerGuide program, and residents’ intentions to participate in the EnerGuide program were 0.734, 0.553, 0.480, and 0.499, respectively. Therefore, the model explained 73.4% of residents’ attitudes toward climate change, 55.3% of their recognition of the benefits of energy efficiency programs, 48.0% of their acceptance of the EnerGuide program, and 49.9% of their intentions to participate in the EnerGuide program. In addition, a measure of approximate fit of the study model, the standardized root mean square residual (SRMR), was 0.052, which was lower than the recommended threshold of 0.080, and the normed fit index (NFI) was 0.879, which was higher than the recommended threshold of 0.800, indicating that the model fit was good (Hair et al., 2014).

**Figure 4:**
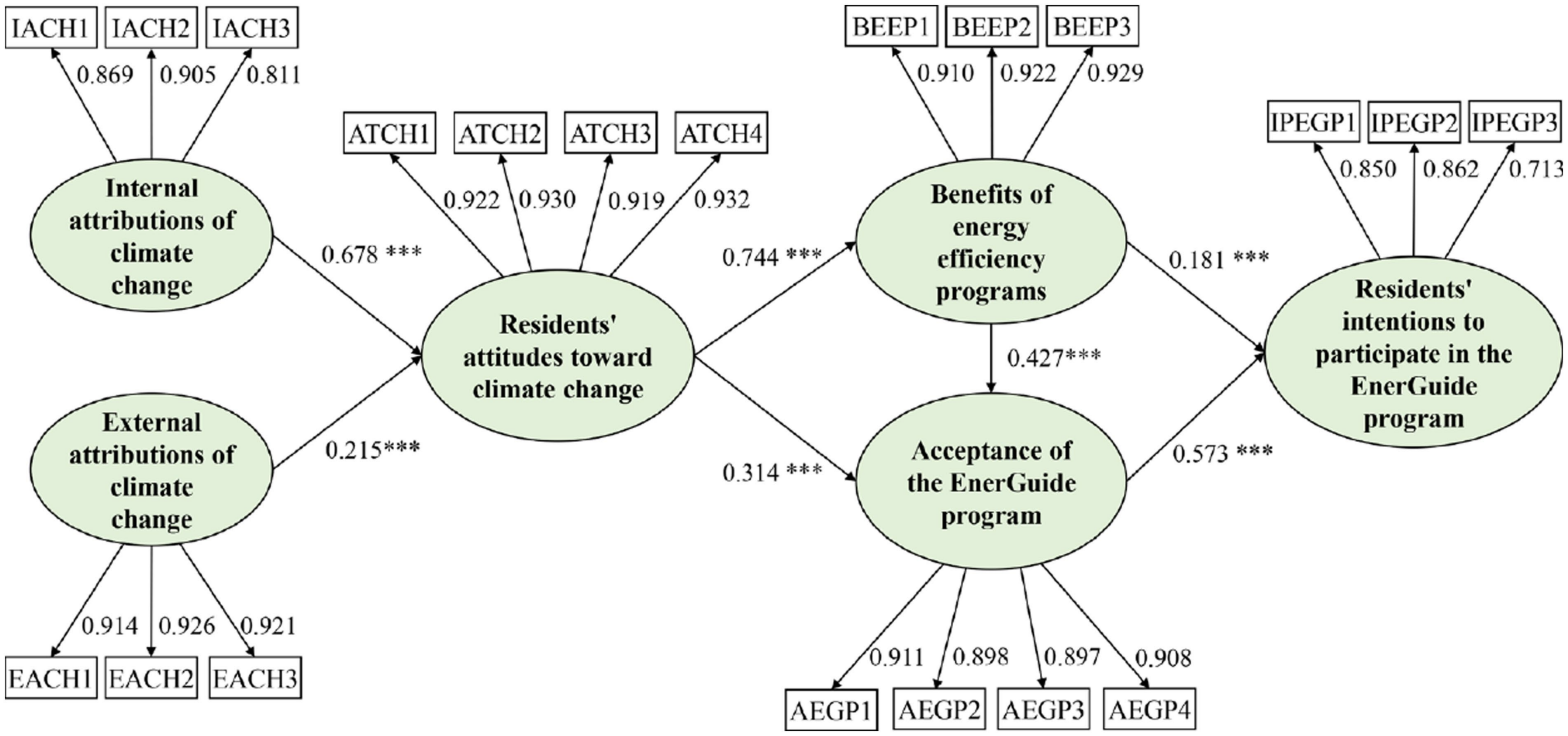
Results of PLS-SEM analysis (***p  <  0.001).

Figure 4 and Table 4 show the relative gravity of the exogenous constructs of residents’ internal attributions, external attributions, climate change attitudes, recognition of the benefits of energy efficiency programs, and acceptance of the EnerGuide program in predicting the endogenous construct of residents’ intentions to participate in the EnerGuide program. Residents’ internal attributions (*β* = 0.678, *p* < 0.001) and external attributions (*β* = 0.215, *p* < 0.001) had a positive impact on their attitudes toward climate change. At the same time, residents’ climate change attitudes had a significant positive impact on their recognition of the benefits of energy efficiency programs (*β* = 0.744, *p* < 0.001) and acceptance of the EnerGuide program (*β* = 0.314, *p* < 0.001). In addition, residents’ recognition of the benefits of energy efficiency programs had a significant positive impact on their acceptance of the EnerGuide program (*β* = 0.427, *p* < 0.001). Residents’ recognition of the benefits of energy efficiency programs (*β* = 0.181, *p* < 0.001) and their acceptance of the EnerGuide program (*β* = 0.573, *p* < 0.001) had a significant positive impact on their intentions to participate in the EnerGuide program.

**Table 4:** Results of hypothesis testing.

Paths	Coefficient	Std. error	*T-value*s	VIF	*f^2^*	Hypothesis	Result
IACH- > ATCH	0.678	0.058	11.763***	2.570	0.672	H1a	Supported
EACH- > ATCH	0.215	0.058	3.689***	2.570	0.068	H2a	Supported
ATCH- > BEEP	0.744	0.029	25.715***	1.000	1.237	H3a	Supported
ATCH- > AEGP	0.314	0.058	5.425***	2.237	0.085	H4a	Supported
BEEP- > AEGP	0.427	0.055	7.816***	2.237	0.157	H5a	Supported
BEEP- > IPEGP	0.181	0.052	3.489***	1.772	0.037	H6	Supported
AEGP- > IPEGP	0.573	0.045	12.825***	1.772	0.370	H7	Supported

***p < 0.001.

In addition, to evaluate whether omitted constructs had a substantive effect on endogenous variables, this study calculated the effect size *f^2^* values (Hair et al., 2017). According to Cohen (1988), the critical values for measuring the effect size *f^2^* are 0.02 (small effect), 0.15 (medium effect) and 0.35 (large effect). As shown in Table 4, the effect size *f^2^* values of the important paths in this study were between 0.037 and 1.237. Therefore, the results of the study showed that the exogenous variables of the structural model had good explanatory power with respect to the endogenous variables.

### 4.3. Mediating effects of attitudes, benefits and acceptance

This study used the bootstrap procedure to analyze the mediating effects of climate change attitudes, the benefits of energy efficiency programs, and acceptance of the EnerGuide program. When testing the mediating effect of residents’ attitudes toward climate change, this study showed that residents’ internal attributions (*β* = 0.213, *p* < 0.001) and external attributions (*β* = 0.067, *p* = 0.005) indirectly influenced their acceptance of the EnerGuide program through the mediating effect of residents’ attitudes toward climate change; this mediating effect was statistically significant at the 5% level, with *t* values of 5.149 and 2.817, respectively. The 95% bias-corrected bootstrap confidence intervals (CIs) of the indirect effects ([LL = 0.136, UL = 0.297] and [LL = 0.029, UL = 0.125]) did not include 0, indicating a mediating effect (Preacher and Hayes, 2008). When testing the mediating effect of residents’ recognition of the benefits of energy efficiency programs, this study showed that residents’ attitudes toward climate change (*β* = 0.317, *p* < 0.001) indirectly influenced their acceptance of the EnerGuide program through the mediating effect of their recognition of the benefits of energy efficiency program; this indirect effect was statistically significant at the 5% level, with a *t-*value of 7.653. The 95% bias-corrected bootstrap CI of the indirect effect [LL = 0.238, UL = 0.398] did not include 0, indicating a mediating effect. Meanwhile, residents’ attitudes toward climate change (*β* = 0.135, *p* = 0.001) indirectly influenced their intentions to participate in the EnerGuide program through the mediating effect of their recognition of the benefits of energy efficiency programs; this indirect effect was statistically significant at the 5% level, with a *t* value of 3.455. The 95% bias-corrected bootstrap CI of the indirect effect [LL = 0.058, UL = 0.210] did not include 0, indicating a mediating effect. In addition, when testing the mediating effect of residents’ acceptance of the EnerGuide program, this study showed that residents’ attitudes toward climate change (*β* = 0.180, *p* < 0.001) indirectly influenced their intentions to participate in the EnerGuide program through the mediating effect of their acceptance of the EnerGuide program; this indirect effect was statistically significant at the 5% level, with a *t* value of 4.732. The 95% bias-corrected bootstrap CI of the indirect effect [LL = 0.110, UL = 0.257] did not include 0, indicating a mediating effect. Meanwhile, residents’ recognition of the benefits of energy efficiency programs (*β* = 0.245, *p* < 0.001) indirectly influenced their intention to participate in the EnerGuide program through the mediating effect of their acceptance of the EnerGuide program; this indirect effect was statistically significant at the 5% level, with a *t* value of 7.130. The 95% bias-corrected bootstrap CI of the indirect effect [LL = 0.181, UL = 0.315] did not include 0, indicating a mediating effect. Therefore, we conclude that there is a mediating effect of residents’ attitudes toward climate change, residents’ recognition of the benefits of energy efficiency programs, and residents’ acceptance of the EnerGuide program (see Table 5).

**Table 5:** Results of mediation analysis.

Relationship	Confidence Interval (BC)						
	Coefficient	Std. error	*T*-values	*P*-values	LL	UL	Result
H1b: IACH- > ATCH- > AEGP	0.213	0.041	5.149	0.000	0.136	0.297	Supported
H2b: EACH- > ATCH- > AEGP	0.067	0.024	2.817	0.005	0.029	0.125	Supported
H3b: ATCH- > BEEP - > AEGP	0.317	0.041	7.653	0.000	0.238	0.398	Supported
H3c: ATCH- > BEEP - > IPEGP	0.135	0.039	3.455	0.001	0.058	0.210	Supported
H4b: ATCH- > AEGP- > IPEGP	0.180	0.038	4.732	0.000	0.110	0.257	Supported
H5b: BEEP- > AEGP- > IPEGP	0.245	0.034	7.130	0.000	0.181	0.315	Supported

BC, bias-corrected; UL, upper level; and LL, lower level.

## 5. Discussion

### 5.1. Key factors influencing residents’ participation intentions

The main factors influencing residents’ intentions to participate in the EnerGuide program have several important theoretical implications. First, the study results indicate that both internal and external attributions of climate change have a significant positive impact on attitudes toward climate change. Compared to external attributions (*β* = 0.215), internal attributions (*β* = 0.678) have a stronger direct influence on attitudes toward climate change. This finding also confirms the perspective of Pivetti et al. (2020), for whom internal attributions are the strongest predictor of attitudes.

Second, the relationship between residents’ attitudes toward climate change and the benefits of energy efficiency programs is positive and significant. Similarly, residents’ attitudes toward climate change also have a positive impact on acceptance of the EnerGuide program. This result means that the level of pro-environmental attitude not only enhances residents’ perception of the benefits of energy efficiency programs but also increases people’s acceptance of the EnerGuide program (Tarigan and Bayer, 2012; Acheampong and Siiba, 2019). The results of this study also confirm that compared to acceptance of the EnerGuide program (*β* = 0.314), climate change attitudes have a greater impact on people’s recognition of the benefits of energy efficiency programs (*β* = 0.744). According to our literature review, no studies have investigated this particular scenario.

Third, the benefits of energy efficiency programs have a significant positive impact on residents’ acceptance of the EnerGuide program – a concept that has not been studied before. This observation suggests that the higher residents perceive the benefits of energy efficiency programs, the more likely they are to accept the EnerGuide program.

Fourth, the benefits of energy efficiency programs and acceptance of the EnerGuide program have a significantly positive impact on residents’ intentions to participate in the EnerGuide program. Similar findings have been noted in previous studies (Syropoulos and Markowitz, 2022; Le-Anh et al., 2023). Compared to the benefits of energy efficiency programs (*β* = 0.181), residents’ acceptance of the EnerGuide program (*β* = 0.573) has a greater influence on their intentions to participate. This study demonstrates that residents’ acceptance of the EnerGuide program greatly affects their intentions to participate. Specifically, the higher the acceptance of the EnerGuide program, the more likely residents are to participate in it.

The conceptual model of climate change discourse established in this study may be an ideal theoretical model for predicting individuals’ intentions to participate in the EnerGuide program and can provide an important theoretical and practical basis for the government to effectively mitigate climate change and improve residential energy efficiency.

### 5.2. Implications for promoting EPCs as a nexus of climate and energy policies

This study highlights that climate and energy policies should be formulated in a two-pronged approach to effectively tackle today’s crises. In this respect, the study of EPCs in the residential sector has important implications.

First, residents’ attitudes toward climate change have an indirect mediating effect on the relationship between the internal/external attribution of climate change and residents’ acceptance of the EPC program. This observation suggests that energy policy can effectively motivate residents’ participation in the EPC program within the discourse of climate change. In other words, the government’s efforts to promote educational programs on climate change and energy shortages can enhance residents’ attitudes toward climate change, thereby increasing their acceptance of the EPC program (Qu et al., 2011).

Second, the benefits of energy efficiency programs have an indirect mediating effect on the relationship between residents’ attitudes toward climate change and acceptance of the EPC program. This result shows that residents’ awareness of the benefits of energy efficiency plays a vital role in linking climate change action and participation in specific energy efficiency programs. The government can use both traditional offline methods, such as public lectures and exhibition boards, and various new media, focusing on a combination of online and offline promotional activities to make residents fully aware of the positive effects of EPC programs on mitigating climate change and reducing residential energy consumption. Notably, the government should highlight in publicity the real benefits brought to residents by energy efficiency programs (Nie et al., 2017; Lau et al., 2020).

### 5.3. Limitations and directions for future research

Although this study provides important theoretical and practical implications by using climate change discourse as the basic theoretical framework, it focuses on intentions rather than outcomes or behaviors. Future research can consider studying actual outcomes, such as the number of households that joined the energy certification program. Meanwhile, the intention–behavior gap has recently been widely discussed in behavioral research in support environments. The proposed model can be used in practice, e.g., in interventions and policy formulation, to bridge this intention–behavior gap. Further work might also consider the role of long-term stable thinking and feeling patterns (e.g., personality traits) in attitudes and intentions. Furthermore, since sociodemographic factors have a broad scope in explaining residents’ environmental protection intentions, future researchers can include more sociodemographic factors such as gender, age, income, educational level, ethnicity and religion.

## 6. Conclusion

This paper developed a theoretical model based on climate change discourse to promote Edmonton residents’ intentions to participate in a local EPC program. The use of individuals’ internal and external attributions to climate change, attitudes toward climate change, and recognition of the benefits of energy efficiency programs can clearly explain residents’ acceptance of the EnerGuide program and their intentions to participate in this program. This study also identified the mediating effects among these variables. First, residents’ attitudes toward climate change have an indirect mediating effect on the relationship between internal/external attributions and acceptance of the EPC program. Second, residents’ attitudes toward climate change have indirect effects on their acceptance of the program and intentions to participate in the program, and this effect is mediated by the benefits of energy efficiency programs. Third, residents’ acceptance of the program has an indirect mediating effect on the relationship between climate change attitudes and intention outcomes. Similarly, residents’ acceptance of the program has an indirect mediating effect on the relationship between the benefits of energy efficiency programs and intention outcomes.

Based on the results of the empirical analysis, this study recommends that the government carry out educational programs on climate change and energy shortages, enrich publicity strategies, and increase subsidy amounts as long-term intervention strategies to promote Edmonton residents’ intentions to participate in the energy efficiency certification program. The findings of this paper regarding climate policy and energy policy complement each other to help government agencies better understand the public’s attitudes toward climate change and perceptions of EPC programs to formulate comprehensive and effective energy and climate change management strategies to reduce residential GHG emissions. This study also provides a clear pathway out of the woods for collective action on climate change in the residential energy sector and makes it easier to build public support for policy action. More significantly, the established climate change discourse framework can be adapted and used in other contexts to understand and encourage local residents’ participation in a range of government energy efficiency ratings and labeling programs.

## Data availability statement

The original contributions presented in the study are included in the article/Supplementary material, further inquiries can be directed to the corresponding author.

## Author contributions

XC: methodology, investigation, data analysis, and writing draft. ZG: supervision and editing draft. HZ: financial support and editing draft. All authors contributed to the article and approved the submitted version.

## Conflict of interest

The authors declare that the research was conducted in the absence of any commercial or financial relationships that could be construed as a potential conflict of interest.

## Publisher’s note

All claims expressed in this article are solely those of the authors and do not necessarily represent those of their affiliated organizations, or those of the publisher, the editors and the reviewers. Any product that may be evaluated in this article, or claim that may be made by its manufacturer, is not guaranteed or endorsed by the publisher.
