# Supplementary material for: Residents’ participation in energy performance certification for collective action on climate change: the case of EnerGuide
Source: Front Psychol. 2023 Jul 11;14:1196208. doi: 10.3389/fpsyg.2023.1196208 (PMC10367088; doi:10.3389/fpsyg.2023.1196208)
Supplement: Supplementary file 1 [file Data_Sheet_1.docx]

# Appendix A. Supplementary data

Supplementary data (City of Edmonton – 2018 Climate Change and Energy Perceptions Survey) for this article can be found online at <https://data.edmonton.ca/Surveys/Climate-Perceptions-Survey-Data-Questionnaire-and-/ueci-2fjj>.

# Appendix B. Climate perception questionnaire

**TABLE B. 1** Climate perception questionnaire.

| Constructs | Items | Question | Mean/  SD* |
| --- | --- | --- | --- |
| Internal attributions of climate change (α=0.828) | IACH1 | Climate change is caused mostly by human activities. | 3.697/  1.194 |
|  | IACH2 | My individual actions contribute to the global impacts of climate change. | 3.658/  1.149 |
|  | IACH3 | My transportation choices impact greenhouse gas emissions. | 3.910/  1.050 |
| External attributions of climate change (α=0.910) | EACH1 | Greenhouse gas emissions contribute to climate change. | 4.100/  0.977 |
|  | EACH2 | Home heating/cooling and electricity use contribute to greenhouse gas emissions. | 3.993/  0.958 |
|  | EACH3 | Workplace heating/cooling and electricity use contribute to greenhouse gas emissions. | 4.027/  0.981 |
| Attitudes toward climate change (α=0.944) | ATCH1 | I am concerned about climate change. | 3.868/  1.120 |
|  | ATCH2 | I think that Edmontonians should be doing more to help prevent climate change. | 3.710/  1.136 |
|  | ATCH3 | I want to do more personally to help prevent climate change. | 3.700/  1.077 |
|  | ATCH4 | I think we need to act now to address climate change. | 3.893/  1.118 |
| Benefits of energy efficiency programs (α=0.910) | BEEP1 | Efforts to prevent climate change present an economic opportunity for Edmonton. | 3.643/  1.093 |
|  | BEEP2 | Transitioning to renewable sources of energy provides job opportunities for Edmonton. | 3.705/  1.130 |
|  | BEEP3 | Investing in energy efficiency provides job opportunities for Edmonton. | 3.765/  1.079 |
| Acceptance of the EnerGuide program (α=0.925) | AEGP1 | Knowing how energy efficient a home is and what needs to be done to make it more efficient would be valuable when searching for homes to buy. | 4.037/  0.875 |
|  | AEGP2 | When buying a home, I would like to see the EnerGuide label. | 3.967/  0.923 |
|  | AEGP3 | The EnerGuide evaluation and expert advice would be valuable to reference when renovating a home. | 4.105/  0.851 |
|  | AEGP4 | Overall, EnerGuide evaluations will provide valuable information to homeowners. | 4.013/  0.853 |
| Intentions to participate in the EnerGuide program (α=0.735) | IPEGP1 | How likely are you to get an EnerGuide home evaluation? | 2.697/  1.373 |
|  | IPEGP2 | How likely is it that you would get an EnerGuide home evaluation if it would allow you to access incentives and rebates to make energy efficiency upgrades in your home? | 3.333/  1.361 |
|  | IPEGP3 | If you were considering purchasing a home and the home inspection indicated inefficiencies in the home’s energy consumption (for example, poor insulation, inefficient appliances, and so forth), how much would this affect your decision to purchase this home? | 3.998/  0.896 |

Note: SD* stands for standard deviation.

# Appendix C. Respondent profile

**TABLE C. 1** Respondent profile.

| Variables | Frequency (n=400) | Percentage (%) |
| --- | --- | --- |
| **Gender**  Male  Female | 209  191 | 52.25%  47.75% |
| **Age**  18-24  25-34  35-44  45-54  55-64  65 or older | 10  74  75  70  95  76 | 2.50%  18.50%  18.75%  17.50%  23.75%  19.00% |
| **Household income before taxes**  Under $40,000  $40,001 to $60,000  $60,001 to $80,000  $80,001 to $100,000  $100,001 to $150,000  More than $150,000 | 50  55  60  73  100  62 | 12.50%  13.75%  15.00%  18.25%  25.00%  15.50% |
| **Highest level of education**  Elementary school  Some high school  Completed high school  Some community college/technical college/CEGEP  Completed community college/technical college/CEGEP  Some university  Completed university  Post-graduate degree  No schooling | 1  5  40  37  89  37  132  59  0 | 0.25%  1.25%  10.00%  9.25%  22.25%  9.25%  33.00%  14.75%  0.00% |
| **Housing type**  Fully detached house  Semidetached house/duplex  Townhouse/row house  Condo/apartment  Other  Not sure | 290  31  28  49  1  1 | 72.50%  7.75%  7.00%  12.25%  0.25%  0.25% |
